# Supplementary material for: Structural basis for mTORC1 activation on the lysosomal membrane
Source: Nature. 2025 Sep 17;647(8089):536–43. doi: 10.1038/s41586-025-09545-3 (PMC12448111; doi:10.1038/s41586-025-09545-3)

nature portfolio

https://doi.org/10.1038/s41586-025-09545-3

Supplementary information

Structural basis for mTORC1 activation on the lysosomal membrane

In the format provided by the authors and unedited

Supplementary Table 1

Lipids			
**Catalog number**	**Product**	**Mol %**	**Company**
850375C	DOPC	72.8	Avanti Polar Lipids, Inc
840034C	POPS	7	Avanti Polar Lipids, Inc
700000P	Cholesterol	10	Avanti Polar Lipids, Inc
790404C	DGS-NTA	5	Avanti Polar Lipids, Inc
780201C	PE-MCC	5	Avanti Polar Lipids, Inc
D12731	DiR	0.2	Invitrogen

Lipids			
**Catalog number**	**Product**	**Mol %**	**Company**
850375C	DOPC	76.3	Avanti Polar Lipids, Inc
850150	PI3P	3.5	Avanti Polar Lipids, Inc
700000P	Cholesterol	10	Avanti Polar Lipids, Inc
790404C	DGS-NTA	5	Avanti Polar Lipids, Inc
780201C	PE-MCC	5	Avanti Polar Lipids, Inc
D12731	DiR	0.2	Invitrogen

Lipids			
**Catalog number**	**Product**	**Mol %**	**Company**
850375C	DOPC	76.3	Avanti Polar Lipids, Inc
850151	PI4P	3.5	Avanti Polar Lipids, Inc
700000P	Cholesterol	10	Avanti Polar Lipids, Inc
790404C	DGS-NTA	5	Avanti Polar Lipids, Inc
780201C	PE-MCC	5	Avanti Polar Lipids, Inc
D12731	DiR	0.2	Invitrogen

Supplementary Figure. 1

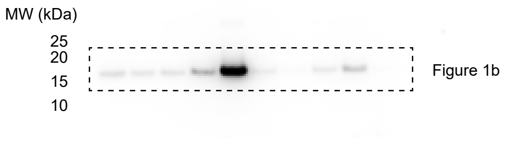

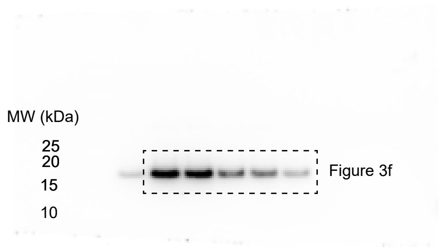

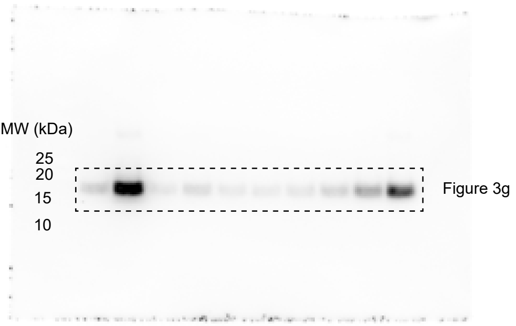

Figure 3h

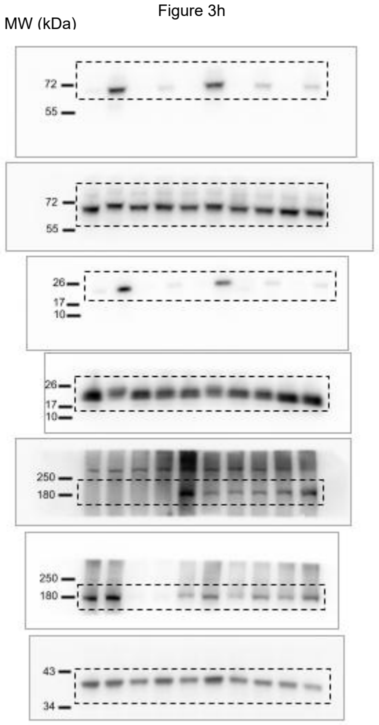

Extended Data Figure 6a

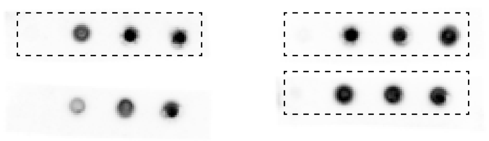

Supplement: Supplementary file 1 — Supplementary Table 1 shows the lipid composition for liposomes used in this study. Supplementary Fig. 1 shows the uncropped images. The cropped regions shown in Figs. 1b and 3f–h and Extended Data Fig. 6a are indicated with dashed lines. For the in vitro kinase assay, the same amount of substrates is used for the reaction. [file 41586_2025_9545_MOESM1_ESM.pdf]
