# Supplementary material for: Structural basis for mTORC1 activation on the lysosomal membrane
Source: Nature. 2025 Sep 17;647(8089):536–43. doi: 10.1038/s41586-025-09545-3 (PMC12448111; doi:10.1038/s41586-025-09545-3)
Supplement: Supplementary file 2 — Reporting Summary [file 41586_2025_9545_MOESM2_ESM.pdf]

nature portfolio

Corresponding author(s): James H. Hurley

Last updated by author(s): Aug 3, 2025

Reporting Summary

Nature Portfolio wishes to improve the reproducibility of the work that we publish. This form provides structure for consistency and transparency in reporting. For further information on Nature Portfolio policies, see our Editorial Policies and the Editorial Policy Checklist.

Statistics

For all statistical analyses, confirm that the following items are present in the figure legend, table legend, main text, or Methods section.

n/a	Confirmed	
☐	☒	The exact sample size (*n*) for each experimental group/condition, given as a discrete number and unit of measurement
☐	☒	A statement on whether measurements were taken from distinct samples or whether the same sample was measured repeatedly
☐	☒	The statistical test(s) used AND whether they are one- or two-sided<br>*Only common tests should be described solely by name; describe more complex techniques in the Methods section.*
☒	☐	A description of all covariates tested
☒	☐	A description of any assumptions or corrections, such as tests of normality and adjustment for multiple comparisons
☐	☒	A full description of the statistical parameters including central tendency (e.g. means) or other basic estimates (e.g. regression coefficient) AND variation (e.g. standard deviation) or associated estimates of uncertainty (e.g. confidence intervals)
☐	☒	For null hypothesis testing, the test statistic (e.g. *F*, *t*, *r*) with confidence intervals, effect sizes, degrees of freedom and *P* value noted<br>*Give P values as exact values whenever suitable.*
☒	☐	For Bayesian analysis, information on the choice of priors and Markov chain Monte Carlo settings
☒	☐	For hierarchical and complex designs, identification of the appropriate level for tests and full reporting of outcomes
☒	☐	Estimates of effect sizes (e.g. Cohen's *d*, Pearson's *r*), indicating how they were calculated

Our web collection on statistics for biologists contains articles on many of the points above.

Software and code

Policy information about availability of computer code

Data collection	SerialEM 4.2
Data analysis	CryoSPARC v4.4, Chimera X 1.6, COOT 0.9, ISOLDE , Phenix 1.21.1, GraphPad Prism 10, and Fiji

For manuscripts utilizing custom algorithms or software that are central to the research but not yet described in published literature, software must be made available to editors and reviewers. We strongly encourage code deposition in a community repository (e.g. GitHub). See the Nature Portfolio guidelines for submitting code & software for further information.

Data

Policy information about availability of data

All manuscripts must include a data availability statement. This statement should provide the following information, where applicable:

- Accession codes, unique identifiers, or web links for publicly available datasets
- A description of any restrictions on data availability
- For clinical datasets or third party data, please ensure that the statement adheres to our policy

Structural coordinates were deposited in the PDB with accession codes 9ED4 (mTORC1-Rag-Ragultor-4EBP1), 9ED6 (mLST8-Rag-Ragultor), 9ED7 (fully active state of mTOR-Rheb), and 9ED8 (Intermediate state of mTOR-Rheb). The cryo-EM density maps were deposited in the Electron Microscopy Data Bank with accession numbers EMD-47932 (a composite map of mTORC1-Rag-Ragultor-4EBP1 on membrane), EMD-47933 (focused refinement of mLST8-Rag-Ragultor subcomplex), EMD-47934 (mTORC1-Rag-Ragulator-4EBP1 on membrane with two extra Rag-Ragulator), EMD-47935 (mTORC1-Rag-Ragulator-4EBP1 on membrane with one extra Rag-Ragulator), EMD-47936 (mTORC1-Rag-Ragulator-4EBP1 complex on membrane with C2 symmetry), EMD-47937 (focused refinement of the mTORC1-Rag-Ragulator-4EBP1 on membrane with mTOR-mLST8-Rheb mask), EMD-47938 (focused refinement of the mTORC1-Rag-Ragulator-4EBP1 on membrane with Raptor-Rag-Ragulator mask), EMD-47939 (fully active state of mTOR on membrane), and EMD-47940 (intermediate state of mTOR on membrane).

Field-specific reporting

Please select the one below that is the best fit for your research. If you are not sure, read the appropriate sections before making your selection.

☒ Life sciences ☐ Behavioural & social sciences ☐ Ecological, evolutionary & environmental sciences

For a reference copy of the document with all sections, see nature.com/documents/nr-reporting-summary-flat.pdf

Life sciences study design

All studies must disclose on these points even when the disclosure is negative.

Sample size	a common practice of 3 replicates for in vitro biochemical assay is chosen
Data exclusions	no data were excluded from the analyses
Replication	all attempts at replication were successful
Randomization	different aliquots of protein samples from different purifications are randomly chosen for biochemical assays.
Blinding	Blinding is not possible because we need to know the exact components of each biochemical assay.

Reporting for specific materials, systems and methods

We require information from authors about some types of materials, experimental systems and methods used in many studies. Here, indicate whether each material, system or method listed is relevant to your study. If you are not sure if a list item applies to your research, read the appropriate section before selecting a response.

Materials & experimental systems

n/a	Involved in the study	
☐	☒	Antibodies
☐	☒	Eukaryotic cell lines
☒	☐	Palaeontology and archaeology
☒	☐	Animals and other organisms
☒	☐	Human research participants
☒	☐	Clinical data
☒	☐	Dual use research of concern

Methods

n/a	Involved in the study	
☒	☐	ChIP-seq
☒	☐	Flow cytometry
☒	☐	MRI-based neuroimaging

Antibodies

Antibodies used	Phospho-p70 S6 Kinase (Thr389) (1A5) (Mouse mAb, Cat# 9206 - 1:1000 WB), p70 S6 Kinase (Rabbit, Cat# 9202 - 1:1000 WB), 4E-BP1 (Rabbit, Cat# 9644 - 1:1000 WB), Phospho-4E-BP1 (Ser65) (Rabbit, Cat# 9456 - 1:1000 WB), Phospho-4E-BP1 (Thr37/46) (Rabbit, Cat # 236B4 - 1:10,000 WB) and Raptor (24C12) (Rabbit, Cat# 2280 - 1:1000 WB) were from Cell Signaling Technology; anti-GAPDH (6C5) (Rabbit, Cat# sc-32233 - 1:15000 WB) was from Santa Cruz; and FLAG M2 (Mouse, Cat# F1804 – 1:1000 WB) was from Sigma Aldrich
Validation	Validations of antibodies used above can be found on manufacture's websites.<br>https://www.cellsignal.com/products/primary-antibodies/phospho-p70-s6-kinase-thr389-1a5-mouse-mab/9206<br>https://www.cellsignal.com/products/primary-antibodies/p70-s6-kinase-antibody/9202<br>https://www.cellsignal.com/products/primary-antibodies/4e-bp1-53h11-rabbit-mab/9644<br>https://www.cellsignal.com/products/primary-antibodies/phospho-4e-bp1-ser65-174a9-rabbit-mab/9456<br>https://www.cellsignal.com/products/primary-antibodies/phospho-4e-bp1-thr37-46-236b4-rabbit-mab/2855<br>https://www.cellsignal.com/products/primary-antibodies/raptor-24c12-rabbit-mab/2280<br>https://www.scbt.com/p/gapdh-antibody-6c5?srsltid=AfmBOoqB9FWmJQBQ42uu9vnfp1jtE696jFx2sRdRMPoyOZugR2rYyYOe<br>https://www.sigmaaldrich.com/US/en/product/sigma/f1804?utm_source=google&utm_medium=cpc&utm_campaign=22180208315&utm_content=179433150172&gad_source=1&gad_campaignid=22180208315&gbraid=0AAAAAD8kLQQX0NQSM-GS9nfwDC6HlIBvo&gclid=CjwKCAjwkbzEBhAVEiwA4V-yqmKnx_hBy6rXA8v95W2p24fbsfKP7X3ZO-jiS71l8GJfV3qGITf7dBoCnGsQAvD_BwE

Eukaryotic cell lines

Policy information about cell lines

Cell line source(s)	HEK293F GnTI is from UC Berkeley cell culture facility (https://bds.berkeley.edu/facilities/cell-culture); Inducible Raptor KO MEFs is a gift from Michael Hall, Univ. of Basel (https://link.springer.com/protocol/10.1007/978-1-61779-430-8_16)
Authentication	Cell lines were validated by morphological analysis
Mycoplasma contamination	Cell lines were routinely tested for absence of mycoplasma.
Commonly misidentified lines (See ICLAC register)	no commonly misidentified cell lines were used in the study
